# Working with bacteria and putting bacteria to work: The biopolitics of synthetic biology for energy in the United Kingdom

**DOI:** 10.1016/j.erss.2017.06.017

**Published:** 2017-08

**Authors:** Carmen McLeod, Brigitte Nerlich, Alison Mohr

**Affiliations:** a University of Oxford, United Kingdom; b University of Nottingham , University of Nottingham, United Kingdom

**Keywords:** Synthetic biology, Bioeconomy, Biopolitics, Responsible research and innovation

## Abstract

The UK government has made significant investment into so called ‘fourth-generation’ biofuel technologies. These biofuels are based on engineering the metabolic pathways of bacteria in order to create products compatible with existing infrastructure. Bacteria play an important role in what is promoted as a potentially new biological industrial revolution, which could address some of the negative environmental legacies of the last. This article presents results from ethnographic research with synthetic biologists who are challenged with balancing the curiosity-driven and intrinsically fulfilling scientific task of working with bacteria, alongside the policy-driven task of putting bacteria to work for extrinsic economic gains. In addition, the scientists also have to balance these demands with a new research governance framework, Responsible Research and Innovation, which envisions technoscientific innovation will be responsive to societal concerns and work in collaboration with stakeholders and members of the public. Major themes emerging from the ethnographic research revolve around stewardship, care, responsibility and agency. An overall conflict surfaces between individual agents assuming responsibility for ‘stewarding’ bacteria, against funding systems and structures imposing responsibility for economic growth. We discuss these findings against the theoretical backdrop of a new concept of ‘energopolitics’ and an anthropology of ethics and responsibility.

## 1 Introduction

It has been forecast that the twenty-first century will be fundamentally influenced by a “Biotechnology Revolution”, in which synthetic biology will play an integral role [1]. In 2008 a headline in a UK newspaper proclaimed “Synthetic biology aims to solve energy conundrum” [2]. For over a decade, many synthetic biologists have aligned their work with this aim through research that modifies enzymes and bacteria in order to produce new (bio)fuels and new (bio)chemicals. The UK government has also made significant investment in so-called “fourth-generation” biofuel technologies [3].

In this context, synthetic biology has become part of a new “biopolitics” [4], where developing novel sources of bioenergy play a key supporting role in the growing bioeconomy. This biopolitical context has become more complicated since 2011, with the promotion of a new science governance framework, Responsible Research and Innovation (RRI).^1^ There is currently no consensus on how RRI should be applied, and it is being operationalised in various ways within different governance and geographical contexts (see [5], [6], [7]). UK academics (e.g. [8]) have introduced RRI as a means of reframing responsibility within innovation as a collective activity that acknowledges the uncertain and political nature of controversial science, focussing on the purpose and possibilities of science and innovation, not just the risks.

The Research Councils UK (RCUK) have included RRI in funding calls, especially in the context of setting up six synthetic biology research centres [9]. Some centres focus on energy and the production of biofuels, and it is hoped that re-engineering particular bacteria will produce microorganisms which can feed on ‘waste’ gases, such as carbon monoxide and carbon dioxide, and also produce biofuels (such as butanol). The UK Government investment in synthetic biology is outlined in the 2012 *Synthetic Biology Roadmap*, which lays out five key recommendations. The third is to “invest to accelerate technology responsibly to market” ([10], p. 31–33). In 2016 the roadmap was reformulated into a strategic plan entitled “Biodesign for the Bioeconomy” [11] with a heavy emphasis on ‘acceleration’ within synthetic biology research.

Based on the results of ethnographic fieldwork, this article considers two challenges faced by synthetic biologists. One challenge is how to balance the curiosity-driven and intrinsically fulfilling scientific process of *working with bacteria* to find out how life works with the task of *putting bacteria to work* in order to achieve extrinsic economic value and generate growth for industry. The other challenge is how to do this within an RRI framework which demands time for reflection and debate. These tensions will be explored by tapping into philosophical reflections on personhood, agency, power and accountability and into anthropological reflections on stewardship [12], which echo some aspects of RRI discourse.

The next section provides a conceptual and theoretical background on the anthropology of energy, “energopolitics” and biofuels, linked to a discussion on the mobilisation of RRI within the context of synthetic biology. Following this, we outline the aims of the article and the methods used in our research. The next substantive results section presents detailed analysis of qualitative interviews and participant observation with a UK synthetic biology research centre, before the final sections which present our discussion and conclusion.

## 2 Conceptual and theoretical background

### 2.1 The anthropology of energy, energopolitics and biofuels

A growing body of social science literature has identified the need for greater attention to the complex socio-political dimensions of energy [13], [14], [15], [16]. Within this, scholarship on the anthropology of energy is building rich and detailed insights into the varied ‘cultures of energy’ and multiple technologies and infrastructures that intersect with energy production, distribution, use (or consumption) and waste (see [17]; and this special issue). In this article, we are particularly interested in the intersection of biology, (bio)politics and (bio)energy with RRI. Biopolitics is defined by Michel Foucault as: “the set of mechanisms through which the basic biological features of the human species became the object of political strategy” ([18], p. 1). Since Foucault, the concept has been used widely and varyingly in social theory to study advances in science and technology that pertain to health and medicine, in particular.

Building on Foucault’s concept of biopolitics, Dominic Boyer’s notion of “energopolitics” provides a useful framework for considering how “the organization and dynamics of political forces across difference scales” manifest themselves in the context of energy ([19], p. 326). Energopolitics highlights the ways in which mechanisms at the macro- and micro- level are entangled. We would extend this entanglement to human-bacteria interactions. In the domain of energy politics, the succession of biofuel ‘generations’ illustrates how the promise of new energy products may ultimately be undesirable when social, ethical or environmental factors are taken into account. For a case study which explores these complex factors in relation to the original biofuel, wood, see Taylor et al. [20] on the politics of conservation, migration and wood-burning in Guatemala.

We would add that biopolitics also refers to the set of mechanisms through which the basic biological features of *bacteria* became the object of political strategy and with those who work with bacteria. In the context of climate change and trying to find new sources of energy, this type of bacterial biopolitics merges with energy politics or “energopolitics”: the set of mechanisms through which the basic choices in energy production and consumption become the object of political strategy.

Each successive generation of biofuels began with promises to ‘save the planet’ or ‘green the planet’. However, each successive generation has eventually come up against major ecological and economic problems [21]. Fourth-generation biofuels are no exception. In 2015, media reported [22] that the synthetic biology biofuels mission had failed. While biofuels produced via the genetic manipulation of algae (supported through over $54 million in loans and backing by the US Government) resulted in the successful development of large amounts of ‘green crude’, a drop in crude oil prices in 2015 and 2016 meant (syn)biofuels could not compete with the economies of scale of oil and gas. This example illustrates how the threat of economic losses can override potential environmental wins. Biopolitics, energopolitics and economics are intricately intertwined. The concatenation of all three produces new conceptual openings for how we think about research governance and related notions of responsibility and agency in complex innovation systems. This is a complicated ‘roadmap’ fraught with ethical potholes that scientists have to navigate with care and responsibility.

Deplazes et al. ([23], p. 66) have identified three potential types of ethical issues related to synthetic biology: “method-related” (ethical questions relating to the ‘moral status’ of the products of synthetic biology); “application-related” (ethical considerations about the potential impacts of future synthetic biology applications); and “distribution-related” (ensuring synthetic biology products are delivered where needed most). This focus on the downstream products and processes of synthetic biology might obscure considerations of the just and responsible treatment of the bacteria that are used to make these products. Bioengineering bacteria for industrial use might raise ethical questions about treating living organisms as machines or tools. In this article we also highlight potential risks to scientists being themselves used as tools in this new biopolitics, risks that are not yet on the horizon of the current RRI agenda.

RRI is a relatively new research governance framework concerned with the nature and trajectory of research and innovation, ensuring that new technologies closely align with societal needs and values. RRI has emerged and evolved in parallel with synthetic biology research and innovation processes. Funders of synthetic biology projects in the UK, such as the EPSRC (Engineering and Physical Sciences Research Council), the BBSRC (Biotechnology and Biological Sciences Research Council) and the European Commission’s Horizon 2020 programme, amongst others, have required that RRI is taken into account within the scientific research.

All six UK synthetic biology research centres funded by the Research Councils are expected to integrate RRI into their research programmes. This implementation is facilitated by embedded social researchers who collaborate with the centres’ scientists and with external stakeholders, including industry and members of the general public who play both agenda-setting and end-user roles in processes of RRI. Four dimensions of RRI have been identified that provide a framework for raising, discussing and responding to social and ethical questions: anticipating intended and unintended impacts; reflection on research motivations, implications, and uncertainties; broad engagement with public and direct stakeholders; and acting on this information to influence research directions [24], [25]. RRI approaches consider broadly how research and innovation could be used in future and the potential impacts that this could have in target markets, but also any indirect effects (e.g., competition for resources; unintended or uneven impacts on different groups or locations) that may arise. In the context of the work undertaken by various synthetic biology research centres around biofuels, these four dimensions are used to inform judgements and practices of ‘energy ethics’. In addition to these four dimensions, stewardship is also an aspect of RRI where responsibility in science and innovation has been defined as ‘taking care of the future through collective stewardship of science and innovation in the present’ ([24], p. 1570) Stewardship, especially environmental stewardship, has a long tradition in ethics and refers to the responsible management of and care for resources – which in our case are not only energy resources, but also bacteria and scientists.

Proponents of RRI are developing new approaches to ethics and responsibility, which are grounded in philosophy, ethics and the social study of science. However, less attention has focussed on theories of stewardship, responsibility and agency, which have long traditions in anthropology [26]. Previous studies with engineers have highlighted how, as agents acting on their own, there is a tendency to shift moral responsibility in techno-scientific innovation to others because of a perception of a lack of agency. Changes to structural characteristics, such as funding rules, may encourage scientists and technologists to embrace issues of broader social responsibility in their work [27]. However, such changes may also lead to loss of agency and rejection of wider responsibilities. A focus on stewardship might help here.

According to anthropologist of ethics, Laidlaw ([28], p. 143–144), agency can refer to the “creative and assertive capacities of individuals, as against the constraints of what are conceived as ‘larger structures’ (discourses, ideologies, cultures, and so on)”. This tension between agents (scientists) and systems or structures (universities, funding councils, industry) is one we shall be exploring further. Laidlaw also reflects on how responsibility is assumed or imposed enhancing or diminishing human agency in the process. In analysing our data we pay particular attention to how responsibility is assumed (by scientists for bacteria and the laboratory) and imposed (by funders on scientists for economic growth and responsible innovation), and what this means for biopolitics – power over biological life – and “energopolitics” – power over (and through) energy [19].

Our work aims to contribute to the small, but growing, social research within the context of synthetic biology e.g. [29], [30], [31]. One previous study, involving anthropologist Paul Rabinow in the US, is rather notorious [32]. Rabinow and his colleague, Gaymon Bennett aimed to foster “coproduction” among multiple disciplines and perspectives from the outset within the frame of “Human Practices”. They developed this term in order to explore the ways in which synthetic biology succeeded (or failed) in meeting promised societal inputs into energy, as well as medicine, security, and the environment. In using participant-observation methods in their research, Rabinow and Bennett understood participant observation to be defined “as both observation and intervention as a mode of inquiry” ([32], p. 6) (drawing on Niklas Luhmann). Their efforts to recalibrate how scientists and social scientists understand themselves and their work within a wider technoscience framework was difficult and has become a “cautionary tale” for others working in this area [33]. Rabinow and Bennett [32] observe that “forms of resistance” to the social research agenda were found at all levels of social organisation and they link the everyday micro-interactions within a synthetic biology research centre, with broader, political and established structures of power influencing scientific research.

We have explicitly chosen to approach our project with *collaboration* as a key element in our research method [31], [34], [35]. Our aim is to discover (together with the scientists) the challenges, as well as the assumed positive outcomes, of implementing RRI within the work of the synthetic biology centre. In the following section we outline the methods in our project in more detail.

## 3 Methods and data analysis

This article draws on material from an ethnographic research project (18 months at time of writing), within a UK synthetic biology research centre. Methods include participant- observation as well as 22 semi-structured base-line interviews with synthetic biology scientists and doctoral students.^2^ We have also incorporated a linguistic focus within our research in order to explore how language use intersects with the application of an RRI framework. The interviews probed participants’ understanding of ethics and responsibility before the embedding of RRI within the research centre. Alongside the interviews and participant observation, the authors carried out an analysis of relevant literature, policy documents, and other relevant material at the intersection of RRI, research governance and science and innovation.

Although ‘bacteria’ were not an explicit focus of our initial interview schedule, it soon became apparent that participants wanted to talk about them (see Introduction to this volume). Accordingly, our analysis also includes consideration of how synthetic biologists grapple with using, understanding and exploiting bacteria in the context of a large research centre tasked to accelerate metabolic engineering of bacteria “responsibly to market” ([10], p. 32).

All interviews were digitally recorded and professionally transcribed verbatim. A thematic analysis of the interview transcripts was carried out using *NVivo,* a software analysis tool, where themes were identified through an inductive process until we were satisfied we had reached data saturation [36], [37]. Thematic analysis has been described as “a method for identifying, analysing and reporting patterns (themes) within data” ([38], p. 78).

Guided by our research questions, we looked for patterns of recurring themes/narratives within our corpus [39]. We paid attention to repeatedly voiced metaphors and analogies. Metaphors enable aspects of more familiar knowledge to be mapped onto more unfamiliar knowledge [40], for example, mapping knowledge about working with people onto knowledge about working with bacteria. By examining pervasive themes and metaphors, we tried to ascertain how synthetic biologists make sense of the work they do in the context of a new biopolitics informed by RRI. These data were then organized in terms of four overarching themes, and eight sub-narratives, which we now discuss.

## 4 Results: themes and sub-narratives

We identified four overarching themes: ‘Bacteria to the rescue: stewardship of the planet’; ‘Putting bacteria to work: practical stewardship’; ‘Working with bacteria: sociable stewardship’; and: ‘Time, scale and agency: tensions in stewardship’. Within these overarching themes and narratives we then searched for sub-narratives that supported these stories. For example, the theme ‘seeing bacteria as tools’ (where knowledge about machines or tools is mapped metaphorically onto bacteria) supports the overarching ‘putting bacteria to work’ theme. Table 1 summarises the themes and sub-narratives we identified in our research – with issues around stewardship and responsibility emerging as a key thread running through the interviews.

**Table 1:** Summary of themes and sub-narratives.

Bacteria to the rescue – stewardship of the planet (Theme 1)	
Sub-narratives:	Examples:
–Saving the planet	“…through biotech technology we are exploring the microorganisms for the future of mankind” [Interlocutor 8].
–Waste to wealth	“so you feed them [bacteria] a waste gas and they produce something valuable” [Interlocutor 1].
Putting bacteria to work practical stewardship (Theme 2)	
Sub-narratives:	Examples:
–Bacteria are tools	“I have never anthropomorphised bacteria. I have always viewed it as being the bag of nuts and bolts” [Interlocutor 17].
–Optimising organisms (for industrial demands)	if I manage to fix all these pathways… that will save you 2.1.p per litre… that’s the goal, that’s the optimisation… an economic benefit [Interlocutor 7].
Working with bacteria – sociable stewardship (Theme 3)	
Sub-narratives:	Examples:
–Bacteria are helpful	“I consider bacteria good, I consider them helpful” [Interlocutor 13]
–Bacteria are sociable	“But when they do [what you want], they make you very happy and very proud” [Interlocutor 16]
Time, scale, and agency – tensions in stewardship (Theme 4)	
Sub-narratives:	Examples:
–The problem of scale	“So if we’re eventually going to make fuel out of waste products, how is that going to affect global politics, and in what way” [Interlocutor 13]
–The pressure of time	“And I think the timelines they have in mind are quite different to the timelines we would think would be appropriate” [Interlocutor 2]

### 4.1 Bacteria to the rescue – stewardship of the planet

#### 4.1.1 Saving the planet

Many of the researchers and doctoral students working on synthetic biology and energy at the Centre appear to be strongly motivated by the hope that their work will make a crucial difference to the environment; that they will contribute to ‘saving the planet’. Many work in the emerging field of ‘green’ or ‘sustainable’ chemistry, an area focused on the design of products and processes that minimize the use and generation of hazardous substances. Chemistry that is, as one scientist described it, “benign by design”. This same scientist went on to say:

I think it [bacteria] is a good thing, because you know through biotech technology we are exploring the microorganisms for the future of mankind [Interlocutor 8].

The most important ambition, shared by all participants, is to move away from an economy and a life-style based on petrochemicals, because as one person commented: “basically everything has come from petroleum….we have to do something about that” [Interlocutor 12]. The same scientist went on to describe the anticipated benefits of the work in the Centre:

...We can’t continue to use fossil fuels, we have to use something else, that’s the major benefit really, reducing pollution and global warming and that’s what we’re trying to do.

The urgency of addressing climate change, therefore, is a key motivation for the scientists in the centre and is considered a “world crisis” [Interlocutor 3]. It is hoped that the work with bacteria will lead to demonstrable benefits to the environment by disrupting the dependence on fossil fuels – ‘breaking the chain’:

We will be able to at, least in part…break that chain or that link between fossil fuels and chemicals at least, and make those chemicals in a sustainable, environmentally friendly way, so that the impact on the environment is much less [Interlocutor 11].

#### 4.1.2 Waste to wealth

Part of this story of ‘bacteria to the rescue’ is that of making them not only contribute to saving the planet, but also to generate wealth or value/valuables from waste. In particular, waste-gases or ‘syngas’, which is a by-product of otherwise polluting steel mills, is central to much of the work being carried out at the Centre.

[Instead of feeding them/organisms sugar] you can basically feed them waste, the carbon monoxide is a waste gas in the industry, so you feed them a waste gas and they produce something valuable. [Interlocutor 1].

There is an implicit tension between gaining valuable insights into the life and work of bacteria and putting them to work to produce ‘valuables’. There is also a tension between gaining knowledge in the present and promising to produce valuable goods and products in the rather distant future.

Many participants were aware that they were making promises about a future based on benign and sustainable fuels and chemicals, which may, in reality, still be a long way off. In talking about the pressure they find themselves under, they used the apt metaphor of ‘reaching boiling point’.

I think a lot of academics feel like they’re under pressure to promise the world, because of your grant… And I think that pressure is building and building and building, and I’m kind of worried it’s going to reach boiling point [Interlocutor 6].

The same participant expressed scepticism about the hype surrounding their work and doubted that bacteria were the “all singing all dancing” panacea policy makers were looking for:

The way that we kind of saw bacteria, was that they were going to do everything, change everything – they were going to be all singing and all dancing… they would be able to create everything… And I suppose what this [PhD] has taught me more, is about the limitations currently in this field [Interlocutor 6].

Some even reflected on the global political context in which they were working and making their promises. They regarded this as rather risky, and more risky than the ‘normal’ risks associated with working with bacteria in their laboratories, or engaging in synthetic biology. They knew how to responsibly handle the normal risks inherent in working with bacteria, but they could not quite envisage how to responsibly handle global, political and economic risks beyond the lab.

And one of the things that I always think about, on a global scale; oil is big business, incredibly big business… So if we’re eventually going to make fuel out of waste products, *how is that going to affect global politics*, and in what way [Interlocutor 13, emphasis added].

And yet, despite being conscious of political risks, scientific limitations, hype and timeframes, there was still hope that the story of bacteria saving the planet might come true, perhaps on a small scale.

I know in the next three years we won’t discover something ground-breaking that will change lives. But hopefully it will make life on this planet more sustainable, help how we deal with it [Interlocutor 2].

In order to deliver on the ‘bacteria to the rescue’ story that structures most of the work that the scientists do within the Centre, bacteria have to be put to work. The Centre’s scientists are therefore under pressure to “make it commercially profitable” [Interlocutor 14] at a larger scale. However, problems associated with scaling-up are causing concerns and anxieties, which some scientists approach with an understanding that bacteria could be a practical ‘tool’ to meet industrial requirements.

### 4.2 Putting bacteria to work – practical stewardship

When putting bacteria to work, scientists begin to see them through a variety of metaphorical or rhetorical lenses. Bacteria become machines or tools that can be coaxed into action and made to perform certain tasks. Above all they can be ‘engineered’ – engineering is the core metaphor structuring the field of synthetic biology (see [41] for a critique of this use of metaphor). As synthetic biology is in effect a new way of genetic engineering and is also linked to systems biology, it is not surprising to find metaphors of systems and engineering here. It is also not entirely unexpected to find that synthetic biology is seen as a way of engineering new products and ‘machines’, as this is part of its official ethos.

#### 4.2.1 Bacteria as ‘tools’

Many participants saw bacteria as tools or ‘nuts and bolts’. They reflected on their usefulness for research and commercialisation.

I have never anthropomorphised bacteria. I have always viewed it [sic] as being the bag of nuts and bolts… I’ve always just viewed it as a tool for the job to be done [Interlocutor 17].

So they’re used as a research tool, they’re hugely important and very beneficial [Interlocutor 11].

Scientists also surprised themselves when thinking about their relationship with bacteria. On the one hand they sometimes anthropomorphised them (working *with* them); on the other they still saw them as tools (putting them to *work*)

Well I do talk to them sometimes, especially when things don’t work. But I think...I treat it quite routinely now, I, oh gosh, I probably do think about them as a tool… [Interlocutor 20].

#### 4.2.2 Optimising organisms (to comply with industrial demands)

In order to make bacteria really useful, scientists have to go beyond bacteria’s natural capabilities and make them better – they need to be optimised to comply with or live up to industrial demands. Optimisation is in fact a frequent talking point amongst PhD students doing their projects within the Centre. In this context, optimisation involves engineering, ‘fixing’, and most, importantly, finding or devising better and more efficient and more useful metabolic pathways. For the following interlocutor, reducing their research to an optimal economic benefit was “depressing”:

This is the depressing bit – if I manage to fix all these pathways that are currently in there, so it doesn’t need any vitamins at all… that will save you 2.1.p per litre… that’s the goal, that’s the optimisation… an economic benefit…as much as possible, for as cheap as possible [Interlocutor 7].

The outcome of this optimisation would, hopefully, be to produce cheaper chemicals in large quantities: “so to make it practical, to make the dream come true, we have to make the bacteria produce a lot more than what it does now” [Interlocutor 7]. Optimisation might not only involve redesigning pathways but also exploiting bacteria’s natural tendency to communicate with each other in the context of what is called quorum sensing. The goal is, again, to increase yields.

I am interested in how the bacteria are communicating, how that works. And from an industry point of view, how it’s going to help industry as a whole, hopefully increase butanol yields, so more car fuel, which is always good (laughs) [Interlocutor 13].

Scientists also have to be careful in choosing which bacteria they use for optimisation and industrialisations because some bacteria are more reticent than others, and do not ‘want’ to be transformed. This finding is consistent with other research carried out with synthetic biologists where the conceptualisation of bacteria is viewed through a lens of how “manipulable” they are (see [31], p. 65). In our research, the ability (or ‘willingness’) of bacteria to be “transformed” emerged as an important theme:

We had a problem with the industrial strain that we were given… in that we could never get that to transform very well… So there’s a very closely related organism… that one you can transform. And it’s quite happy to be transformed [Interlocutor 7].

As Balmer et al. have elaborated: “a defining feature of this emerging techno-science [synthetic biology] is the manipulation of the bacterial world […], and thus also a desire to control the supposedly unruly life of microbes in a more industrially advantageous manner” ([31], p. 59–60). However, this desire may come into conflict with a more deep-seated desire of doing good science and finding things out about the world, as will be explored further in the following section.

### 4.3 Working with bacteria – stewardship on the ground

There is an inherent tension, then, between putting bacteria to work for the sake of commerce on the one hand and working with bacteria for the sake of understanding how life works on the other. Seeing them as ‘machines’ seems to be necessary in both cases – in order to make them function in certain ways for extrinsic gain and in order to gain an intrinsic understand of how they function.

#### 4.3.1 Bacteria are helpful

Despite supporting the overarching narrative that the goal of the Centre is to save the planet and to produce wealth from waste, many interlocutors also voiced a wish to still engage in basic curiosity-driven research. One theme explored by many of our interlocutors was how they viewed bacteria as creatures that were remarkable and deserving of respect. The “helpful” (to human) qualities of bacteria were particularly appreciated and this understanding did not chime so much with framing them as mere machines or tools used in the race to produce a new energy source or other chemicals. Here scientists make sense of bacteria by using, or metaphorically mapping, familiar knowledge or familiar norms and expectations surrounding living and working with people onto bacteria. The following quote exemplifies this understanding:

I consider bacteria good, I consider them helpful. A vast majority of bacteria are not harmful, and in fact causing harm to humans is incredibly difficult for bacteria to do [Interlocutor 13].

This understanding of bacteria as helpful also means thinking about them in the context of the overarching ‘saving the planet’ narrative. They are not enemies; they are friends and co-workers in the scientists’ bid to make the world a better place:

I am a terrible person for anthropomorphisation. I sing to my bacteria to try to get them to grow. Mine are the good guys obviously; they’re there to try to save the world; they’re there to try and reduce our dependence on oil; they’re there to take… greenhouse gases out of the atmosphere. So they’re definitely the good guys [Interlocutor 7].

Closely intertwined with this sub-theme of helpful bacteria was the highlighting of bacteria as social creatures. Accordingly, they not only deserve respect, but also affection.

#### 4.3.2 Bacteria are sociable

The following scientist describes his affection for bacteria in the context of the sociability of bacterial populations:

It’s probably a strange affection. I am always fascinated with bacteria … I really like them. … At the same time, I find it interesting that bacteria are not single isolated cells, but they have social lives. They interact and coordinate behaviour and so on… [Interlocutor 2].

Another view is that bacteria are not just there to be exploited for profit but to be nurtured like ‘children’.

So if I clone something, or if I want them to make something, I have to select individual, babies, I call them my babies… And when they are not doing anything happy…most of the time it’s frustrating, because most of the time they don’t do what you want. But when they do, they make you very happy and very proud [Interlocutor 16].

For scientists on the ground the most important aspect of their work is care, stewardship, responsibility for ‘their’ bacteria in the present, which involves giving these bacteria due respect. They also care about the near future in terms of finishing their PhDs and post-doctoral studies. This care of the personal present is linked by many to care of ‘the planet’. They co-opt their bacteria in their efforts to carry out science that is socially beneficial; that is in principle, they engage in RRI even without using the term. This science stewardship in the laboratory operates at a broader level, incorporating elements of anticipation, reflection, engagement, and responsiveness to varying degrees. We also see stewardship operating literally at the micro-level, where scientist make efforts to responsibly steward the bacteria they work with towards responsible and environmentally beneficial goals.

### 4.4 Time, scale, and agency – tensions in stewardship

It seems that scientists are comfortable working at the human scale, both in terms of space and time. In terms of scale with relation to space, they assume control and responsibility over their labs and their bacterial and human inhabitants. In terms of scale with relation to time, they carefully manage the time spent in the laboratory and with the bacteria and lavish as much time as possible on research and teaching activities. However, there is some anxiety when it comes to the global scale and to speed and acceleration. Can one assume responsibility for global economics and politics which impact on how work with bacteria pans out? And can one accommodate and be responsible for ever-increasing demands to bring research and innovation to fast and speedy fruition?

#### 4.4.1 The problem of scale

One participant was concerned about how the work in the Centre was actually going to make an impact on a bigger scale: “So if we’re eventually going to make fuel out of waste products, *how is that going to affect global politics*, and in what way” [Interlocutor 13, emphasis added]. There is a tension then between taking responsibility at human scale (the laboratory, research centre, etc.) and taking responsibility for the global scale. This tension is exacerbated by demands to scale up production of green energy and become a global competitor in the production of biofuels. There is a lot of pressure to go beyond the human scale, which causes anxiety, as this scale is mostly beyond people’s control.

I know a lot of people, particularly… with their partnership with [industry partner] are under huge amounts of pressure to show where the funding has gone; to show that they can do something [Interlocutor 13].

#### 4.4.2 The pressure of time

The problem of scale is also linked to the issue of time. Social theorists have paid relatively little attention to the intersection of scientists’ personal lives with their professional duties, and Balmer et al. ([31], p. 170) suggest there needs to be more acknowledgement of “the failures, preliminary runs, aggravations, breakdowns, financial difficulties, family and time pressures”, which impact on laboratory work. Taking this further, we argue that more attention should be paid to time pressure which is a topic that looms large in many conversations we have with synthetic biologists. [For an emerging strand of research on the ‘Accelerated Academy’, see [42]]. For example, the following scientist commented on the different timelines between research and the expected delivery of outputs:

I think now there is additional pressure because the people who have provided the money, in the end the government, they want to see some sort of success. And I think the timelines they have in mind are quite different to the timelines we would think would be appropriate, you know, because we know how sometimes the research goes… [Interlocutor 2].

In addition to dealing with the pressure to produce immediate results in the near future, scientists also have to grapple with time in the sense of a promised distant ‘future'. Our interlocutors expressed worries about how they could maintain support from the general public for the research they do and how this support could be undermined by trying to oversell or overhype the promises of putting bacteria to work for a better and greener world. In a sense they fear that they have to use the future to sell the present (see [43]). The following two quotes elaborate on this:

And it’s probably one of the hardest things to communicate to the public, when they …say how does this help, we have to say well it will help, potentially, but selling it on future promises I guess [Interlocutor 13].

If the public aren’t with us, aren’t with scientists, then all this technology falls on its face. And rather than have the promise to make things sustainably greener, environmentally friendly, we get stuck with fossil fuels, or we get stuck with not having resources for a growing population [Interlocutor 11].

Overall, scientists are grappling with tensions and responsibilities in time and space (scale), especially trying to think about global impacts and implications, anticipating future constraints and concerns, and, last but not least, overselling the present by creating imaginary futures of wealth and sustainability.

To this mixture of worries we have to add RRI. While time taken to work with bacteria in curiosity-driven research is valued, and while time taken to put bacteria to work for economic growth is accepted as part of the job, scientists tend to find it difficult to add what one might call ‘RRI time’ to these demands. Doing RRI means pausing and taking time for reflection and anticipation as well as taking time to talk to stakeholders and members of the public and, in an ideal world, change and modify the research process accordingly. This novel demand on time-management is difficult to accommodate within traditional research and innovation processes.

## 5 Discussion

Our analysis has revealed how scientists respond to the tensions and challenges of working in synthetic biology, in particular around the themes of stewardship, care, responsibility and agency. While scientists may want to ‘save the world’, they find it more difficult to create ‘wealth from waste’; while they love and value working with their bacteria and finding things out about them, they find it more problematic to put bacteria to work to create economic value; and while they readily assume responsibility for what they do in the lab, they find it harder to assume responsibility for the world at large.

They also express three key anxieties related to these overall tensions: (1) time pressure to produce marketable products, while at the same time reflecting on short-term and long-term risks and responsibilities; (2) the problem of scaling up from the laboratory to the factory in a global energy market beyond researchers’ control; and (3) the perceived dilemma for researchers of engaging in public dialogue without overselling what they can do and without invoking the ‘spectre’ of another GM controversy [44].

This third anxiety links to an assumption made by some scientists and policy makers that members of the public will be fearful of synthetic biology. This anxiety has been called “synbiophobia-phobia” [45], and social scientists and anthropologists have been asked to help mitigate the potential ‘barriers to innovation’ which might come from public opposition to synthetic biology (e.g. [46]).

Overall then, scientists have to balance the essentially curiosity-driven and intrinsically fulfilling process of working with bacteria, with the essentially more fraught and challenging task of putting bacteria to work towards meeting accelerated expectations to generate growth and wealth. These tensions also find expression in the use of metaphors, where bacteria are conceptualised as machines (in order to understand their functions), as machines or tools (useful for creating wealth) and as personified agents (that deserve care and respect). Bacteria are, therefore, lifeforms that can be controlled, manipulated and transformed, yet “simultaneously appear as vulnerable and as at risk” ([31], p. 64).

The requirement from UK funding organisations to incorporate RRI principles within synthetic biology centres across the UK suggests a new landscape of scientific responsibility where income-generating aspirations are conjoined with social and ethical considerations. However, the relationship between the human (scientist) and non-human (bacteria) is largely ignored in this landscape, as well as the status of the scientist as responsible agent with limited powers. It becomes important in the context of synthetic biology for both human and non-human “to be understood by virtue of their contextual relations with each other” ([31], p. 62). Many scientists articulate a feeling of responsibility towards the bacteria they work with, along with wishing to responsibly put bacteria to work for the benefit of wider society. Yet the framing of responsible science and how it should be mobilised, are not necessarily the same at the laboratory level and the policy level.

There are some difficulties, therefore, in responsibly shaping the present and the future, the personal and the global. There is an assumption that scientists have unfettered agency and power to embed RRI principles within their work. However, as Laidlaw ([47], p. 163) articulates, agency should not be thought of as a “capacity for efficiency inherent in individuals or derived from their subjectivity”, rather it is a “matter of relations that reach both into and beyond the individual”. Scientists can control, to some extent, how they use bacteria for research, what to do with them, what to do for them and so on – they have power and agency in this context. Control and agency become more difficult the further away scientists get from their bacteria and labs. They have no control or power, for example, over the world economy, oil prices and so on, which impinge on the success of fourth generation biofuels – saving the planet and over generating growth and wealth. They have control over basic science and safety procedures in the laboratory, but find it more difficult to demonstrate control over ethical responsibilities to funders, industry, and in the context of the global economy (see [27]).

## 6 Conclusion

RRI is based on the notions of responsibility, care and stewardship. Scientists working in synthetic biology take care of bacteria and assume control and responsibility for their work and the safety of other humans and non-humans in the context of their labs, but also in the context of their ambition to create a greener future for the planet. It is here that they have individual agency and ambition and can act as ‘stewards’. However, their work is also controlled by various agendas, structures and systems that are beyond the control of scientists themselves, such as the growth-agenda and the RRI agenda, which we argue has been largely co-opted into the growth agenda. Surprisingly, the policy makers setting these agendas do not assume the role of stewards themselves. They are not taking collective responsibility for the people that they ‘put to work’. They also mainly overlook how calls for scaling up and promissory discourses of profitable futures create tensions and conflict with the ethos and methods of science and scientists. Policy makers who impose a responsible innovation agenda and an agenda of stewardship on scientists need to also assume responsibility for stewardship of the people they deploy.

The basic conflict in this new RRI infused biopolitical landscape is between power and responsibility, between individual agents assuming responsibility and collective policy bodies and larger structures imposing responsibility. The scientist as ‘responsible agent’ disappears from view in this conflict, as well as the bacteria the scientist works with, and sometimes anthropomorphises as agents, for whom they have responsibility. RRI should empower scientists to widen their circle of responsibility in terms of not only working with bacteria but also with stakeholders and the general public. How this can be achieved in a biopolitical climate dominated by a growth agenda is not entirely clear. It remains to be seen how the expectations generated by RRI itself can be managed in the future and how they can be incorporated into the work of scientists and the work they make bacteria perform.

The tensions associated with engineering bacteria are illustrative of the complex interplay between agency, structure and power in the context of energopolitics. Our findings highlight the key role for anthropologists and social scientists to facilitate discussions about the unevenness of agency and power – or as Andy Stirling terms it “asymmetrically structured agency” ([15], p. 84). In the case we have outlined in this article, it is clear there is an unevenness in the power that scientists have within the agenda set by government – both in the context of doing ‘science’ and in the context of doing ‘responsible research and innovation’. Social scientists and anthropologists working with scientists in the same space can facilitate conversations and interactions that bring tensions into the open, thus laying the groundwork for a better management of scientific, economic and RRI expectations.

## Footnotes

1 The terms RRI and ‘responsible innovation’ are often used interchangeably. Following the lead of the 2012 *UK Roadmap for Synthetic Biology*[10] and to focus attention on the important role of researchers as ‘responsible agents’, we use RRI for the purposes of this article.

2 Ethical approval was sought and granted by the School of Sociology and Social Policy at University of Nottingham.

## References

[1] Elfick A., Endy D., Ginsberg Alexandra Daisy, Calvert Jane, Schyfter Pablo, Elfick Alistair, Endy Drew (2014). ‘Introduction: how would you design nature’. Synthetic Aesthetics: Investigating Synthetic Biology’s Designs on Nature.

[2] Edwards C. (2008). Synthetic biology aims to solve energy conundrum. The Guardian.

[3] Aro E.-M. (2016). From first-generation biofuels to advanced solar biofuels. Ambio.

[4] Prozorov S., Rentea S. (2016). The Routledge Handbook of Biopolitics.

[5] De Saille S. (2015). Innovating innovation policy: the emergence of ‘Responsible Research and Innovation’. J. Responsib. Innov..

[6] Ribeiro B.E., Smith R.D.J., Millar K. (2016). A mobilising concept? Unpacking academic representations of Responsible Research and Innovation. Sci. Eng. Ethics.

[7] Rip A. (2014). The past and future of RRI. Life Sci. Soc. Policy.

[8] Owen R., Macnaghten P., Stilgoe J. (2012). Responsible research and innovation: from science in society to science for society, with society. Sci. Public Policy.

[9] EPSRC (2013). Multidisciplinary Synthetic Biology Research Centres (SBRCs). https://www.epsrc.ac.uk/funding/calls/multidisciplinarysbrcs/.

[10] UK Synthetic Biology Roadmap Coordination Group (2012). Synthetic Biology Roadmap for the UK.

[11] Synthetic Biology Leadership Council (2016). Biodesign for the Bioeconomy: UK Synthetic Biology Strategic Plan 2016.

[12] Pellé S., Reber B. (2015). Responsible innovation in the light of moral responsibility. J. Chain Netw. Sci..

[13] Rogge K.S., Riechardt K. (2016). Policy mixes for sustainability transitions: an extended concept and framework for analysis. Res. Policy.

[14] Sovacool B., Sovacool Benjamin (2013). Introduction. Energy and Ethics: Justice and the Global Energy Challenge.

[15] Stirling A. (2014). Transforming Power: social science and the politics of energy choices. Energy Res. Soc. Sci..

[16] Silvast A., Hänninen H., Hyysalo S. (2013). Guest Editorial. Energy in society: energy systems and infrastructures in society. Sci. Technol. Stud*.*.

[17] Strauss S., Rupp S., Love T., Sarah Strauss, Rupp Stephanie, Love Thomas (2013). Powerlines: cultures of energy in the twenty-first century. Cultures of Energy: Power, Practices, Technologies.

[18] Foucault M., Davidson Arnold I., Burchell Graham (2009). Security, territory, population. Trans. Graham Burchell, Lectures at the Collège de France, 1977–1978.

[19] Boyer D. (2014). Energopower: an introduction. Anthropol. Q..

[20] Taylor M., Moran-Taylor M., Castellanos E., Elías S. (2011). Burning for sustainability: Biomass energy, international migration, and the move to cleaner fuels and cookstoves in Guatemala. Annals of the Association of American Geographers.

[21] Mohr A., Raman S. (2013). Lessons from first-generation biofuels and the implications for sustainability appraisal of second-generation biofuels. Energy Policy.

[22] Ferry D. (2015). The promises and perils of synthetic biology. Newsweek.

[23] Deplazes A., Ganguli-Mitra A., Biller-Andorno N., Schmidt Markus, Kelle Alexander, Ganguli-Mitra Agomoni, de Vriend Huib (2009). The ethics of synthetic biology: outlining the agenda. Synthetic Biology: The Technoscience and Its Societal Consequences.

[24] Stilgoe J., Owen R., Macnaghten P. (2013). Developing a framework for responsible innovation. Res. Policy.

[25] Owen R., Stilgoe J., Macnaghten P., Gorman M., Fisher E., Guston D., Owen Richard, Bessant John, Heintz Maggy (2013). A framework for responsible innovation. Responsible Innovation: Managing the Responsible Emergence of Science and Innovation in Society.

[26] Pellé S., Reber B. (2016). From Ethical Review to Responsible Research and Innovation.

[27] Swierstra T., Jelsma J. (2006). Responsibility without moralism in technoscientific design practice. Sci. Technol. Hum. Values.

[28] Laidlaw J. (2014). The Subject of Virtue: An Anthropology of Ethics and Freedom.

[29] Molyneux-Hodgson S., Meyer M. (2009). Tales of emergence: synthetic biology as a scientific community in the making. BioSocieties.

[30] Finlay S. (2013). Engineering biology? Exploring rhetoric, practice, constraints and collaborations within a synthetic biology research centre. Eng. Stud. (Special Issue on Synthetic Biology).

[31] Balmer A., Bulpin K., Molyneux-Hodgson S. (2016). Synthetic Biology: A Sociology of Changing Practices.

[32] Rabinow P., Bennett G. (2012). Designing Human Practices: An Experiment with Synthetic Biology.

[33] Balmer A.S., Bulpin K. (2013). Review of Paul Rabinow and Gaymon Bennett ‘Designing human practices: an experiment with synthetic biology’. TECNOSCIENZA Ital. J. Sci. Technol. Stud..

[34] Calvert J., Martin P. (2009). The role of social scientists in synthetic biology. EMBO Rep..

[35] Balmer A., Calvert J., Marris C., Molyneux-Hodgson S., Frow E., Kearnes M., Bulpin K., Schyfter P., Mackenzie A., Martin P. (2016). Five rules of thumb for post-ELSI interdisciplinary collaborations. J. Responsib. Innov..

[36] Saumure K., Given L.M., Given L.M. (2008). Data saturation. The SAGE Encyclopedia of Qualitative Research Methods.

[37] Guest G., MacQueen K., Namey E. (2012). Applied Thematic Analysis.

[38] Braun V., Clarke V. (2006). Using thematic analysis in psychology. Qual. Res. Psychol..

[39] Riessman C.K. (2008). Narrative Methods for the Human Sciences.

[40] Lakoff G., Johnson M. (1980). Metaphors We Live By.

[41] Boudry M., Piglucci M. (2013). The mismeasure of machine: synthetic biology and the trouble with engineering metaphors. Stud. Hist. Philos. Sci. Part C: Stud. Hist. Philos. Biol. Biomed. Sci..

[42] Carrigan M. (2015). Life in the Accelerated Academy: Anxiety Thrives, Demands Intensify and Metrics Hold the Tangled Web Together. http://blogs.lse.ac.uk/impactofsocialsciences/2015/04/07/life-in-the-accelerated-academy-carrigan/.

[43] Brown N. (2003). Hope against Hype—accountability in biopasts, presents and futures. Soc. Stud. Sci..

[44] Nerlich N., McLeod C. (2016). The dilemma of raising awareness responsibly. EMBO Rep..

[45] Marris C. (2015). The construction of imaginaries of the public as a threat to synthetic biology. Sci. Cult..

[46] Balmer A., Molyneux-Hodgson S. (2013). Bacterial cultures: ontologies of bacteria and engineering expertise at the nexus of synthetic biology and water services. Eng. Stud..

[47] Laidlaw J., Lambek Michael (2010). Agency and responsibility: perhaps you can have too much of a good thing. Ordinary Ethics: Anthropology, Language, and Action.

